# Responses to Threat of Influenza A(H7N9) and Support for Live Poultry Markets, Hong Kong, 2013

**DOI:** 10.3201/eid2005.131859

**Published:** 2014-05

**Authors:** Peng Wu, Vicky J. Fang, Qiuyan Liao, Diane M.W. Ng, Joseph T. Wu, Gabriel M. Leung, Richard Fielding, Benjamin J. Cowling

**Affiliations:** School of Public Health, The University of Hong Kong, Hong Kong, China

**Keywords:** avian influenza A(H7N9), influenza, viruses, Hong Kong, public health, behavioral response, psychological response, community, H7N9, survey, respiratory infections, live poultry markets, poultry, birds

## Abstract

We conducted a population survey in Hong Kong to gauge psychological and behavioral responses to the threat of influenza A(H7N9) and support for closure of live poultry markets. We found low anxiety and low levels of exposure to live poultry but mixed support for permanent closure of the markets.

A novel influenza A(H7N9) virus was detected in China in March 2013, and an epidemic of infections in poultry and humans occurred during April and May of that year (*1*,*2*). Most persons who had laboratory-confirmed infections reported recent contact with live poultry, and evidence suggests that human-to-human transmissibility of the virus is low (*2*). Incidence of laboratory-confirmed human cases dramatically decreased following the closure of live poultry markets (LPMs) in affected cities in April 2013 (*3*). Control of the virus is challenging because its low pathogenicity in poultry (*4*) requires reliance on laboratory-based surveillance in animals or humans to identify areas where the virus is prevalent and to facilitate interventions to reduce human exposure to infected poultry.

Hong Kong imports live poultry from mainland China only from a few dedicated farms that have adequate biosecurity protection; intensive surveillance for avian influenza is conducted at the border and within Hong Kong. Risk for influenza A(H7N9) virus infection appears to be low in Hong Kong, but 4 infections have been reported in Hong Kong residents since December 2013, and a surge in influenza virus transmission was anticipated in eastern China this winter (*2*). Prevention and control activities rely on accurate measures of exposure to live poultry, risk perception and psychological and behavioral responses related to the virus, and attitudes toward specific control measures. We therefore conducted a series of cross-sectional population surveys to monitor these variables in Hong Kong.

## The Study

We initiated the first survey in April 2013 (April 10–13 and 25–27), shortly after the first human case of influenza A(H7N9) was announced in mainland China. A second survey was conducted December 4–8, after incidence of human cases began to rise in the winter and the first local infection occurred in Hong Kong. We used methods and survey instruments similar to those used for surveys during the severe acute respiratory syndrome epidemic in 2003 (*5*), the influenza A(H1N1)pdm09 pandemic (*6*), and the emergence of avian influenza A(H5N1) (*7*).

For the survey, trained interviewers made telephone calls to land lines by using a computerized, random-digit dialing system; calls were placed during nonworking hours and weekends to avoid overrepresentation of nonworking groups. Within households, adults >18 years of age who spoke Cantonese were eligible and were randomly selected on the basis of a Kish grid (*8*). Up to 4 follow-up calls were made if participants were not available or if calls were unanswered. Verbal informed consent was obtained from all participants. Means and proportions of survey items were directly weighted by sex and age to the general population, and multiple imputation with 10 datasets was used to correct for missing data.

**Figure 1:**
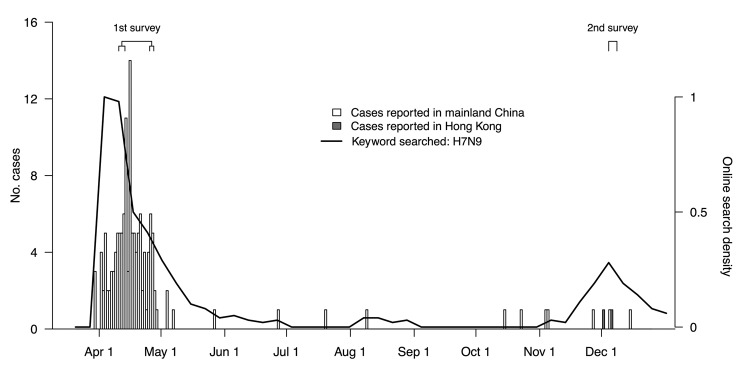
Laboratory-confirmed human cases of influenza A(H7N9) virus infection in mainland China and Hong Kong, by date of announcement, compared with timing of population surveys and public interest in influenza A(H7N9), 2013. Public interest was calculated by using Google Trends (www.google.com/trends) on the basis of internet searches on the keyword H7N9 measured by normalized relative search volume; lines show the ratio of weekly search volume on the defined keywords divided by the search volume on any keyword during the period, after normalizing the highest ratio as 1. April survey was conducted in 2 phases; information was combined for these analyses.

We completed 1,556 interviews during the April survey and 1,000 interviews during the December survey; response rates were 68.9% and 68.0%, respectively. The characteristics of respondents were similar for each survey period (Technical Appendix). Figure 1 illustrates the timeline of laboratory-confirmed cases of influenza A(H7N9) compared with the dates of our surveys.

Table 1 shows a summary of the population anxiety and risk perception related to influenza A(H7N9) across the 2 survey periods. The level of general anxiety in the population, measured by the State Trait Anxiety Inventory (*5*,*6*), remained low and was comparable to population anxiety during April–May 2009 (*6*). Most (85%–89%) respondents identified close contact with chickens in an LPM as a risk factor for infection with influenza A(H7N9) virus. Respondents reported perceived susceptibility of infection as low, perceived severity of influenza A(H7N9) as lower than that of severe acute respiratory syndrome, and perceived severity of influenza A(H7N9) as higher than that of influenza A(H5N1) and seasonal influenza. Respondents also expressed that they would experience low levels of worry if influenza-like symptoms were to develop in the respondent the day after the survey (symptom-induced worry).

**Table 1:** Generalized anxiety and risk perception among persons surveyed during influenza A(H7N9) epidemic, Hong Kong, April and December 2013*

Category	First survey	Second survey	p value
Population anxiety†	1.81	1.79	0.35
Risk perception, %			
Susceptibility‡			
Absolute	12.1	9.3	0.01
Relative	2.3	1.3	0.19
Severity compared with seasonal influenza§	88.1	88.3	0.80
Severity compared with SARS§	39.5	28.8	<0.01
Severity compared with influenza A(H5N1)§	79.1	81.6	0.10
Symptom-induced worry¶	44.8	37.3	<0.01

*First survey conducted April 10–13 and 25–27, 2013; second survey conducted December 4–8, 2013. p values were estimated by comparing anxiety and risk perception between the 2 surveys after adjustment for demographics including age, sex, education, place of birth, marital status, and household income. SARS, severe acute respiratory syndrome. †Measured by 4-point State Trait Anxiety Inventory (*5*; 1 indicates least anxiety, 4 most anxiety).  ‡Absolute susceptibility was examined by asking how likely the survey participant thought it was that he or she would contract influenza A(H7N9) during the next month; relative susceptibility was examined by asking how likely the survey participant thought it was that he or she would contract influenza A(H7N9) during the next month compared with persons outside his or her family with similar age. Answers were given on a 7-point scale and measured as proportion of respondents whose answer was likely, very likely, or certain. §Perceived severity was examined by asking respondents how the severity of influenza A(H7N9) compared with that of seasonal influenza, SARS, and influenza A(H5N1). Answers were given on a 5-point scale and measured as proportion of respondents whose answer was either a bit higher or much higher. ¶Perceived anxiety level if respondent were to experience onset of influenza-like symptoms in the next day. Answers were given a 7-point scale and measured as proportion of respondents whose answer was worried more than normal, worried much more than normal, or extremely worried.

**Figure 2:**
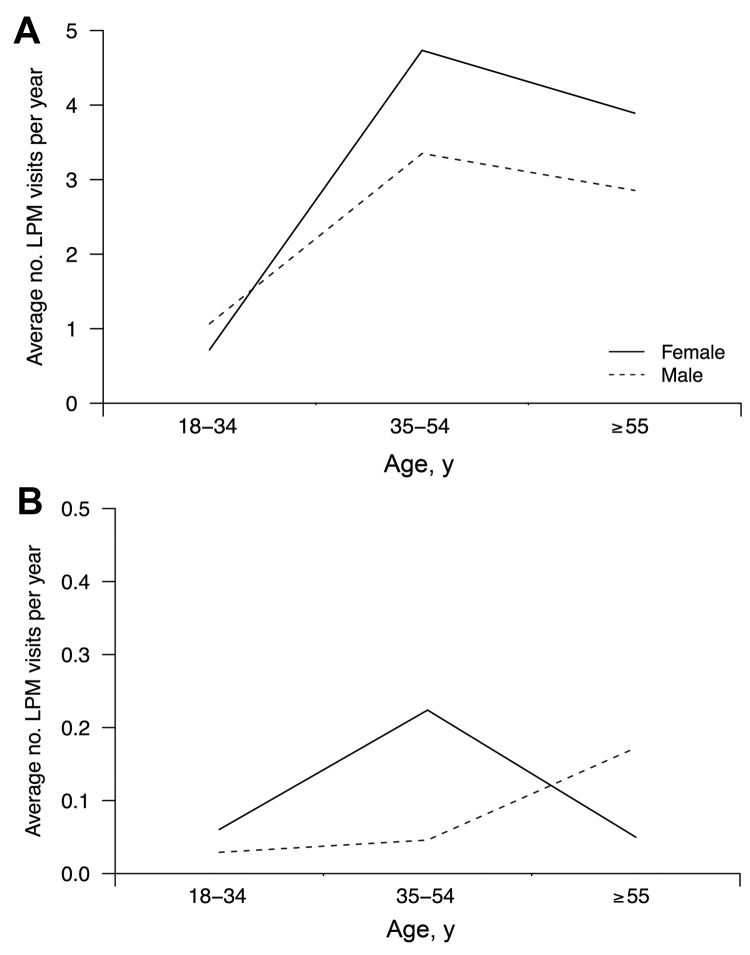
Exposures to live poultry among Hong Kong residents, by age and sex, in terms of weighted average numbers of visits per year to live poultry markets in Hong Kong (A) and mainland China (B).

During the December survey, we also collected data on exposures to live poultry markets (LPMs). A total of 26.7% of respondents reported visiting an LPM in Hong Kong >1 time during the previous year; of those, 61.4% and 37.5% reported visiting >1 time per month and >1 time per week, respectively. In addition, 6.9% of respondents reported visiting an LPM in mainland China >1 time during the previous year. Across the population, we estimated the average numbers of annual visits to LPMs in Hong Kong and mainland China to be 17.6 and 0.53 visits per person, respectively. These estimates were based on an assumption of standardized annual numbers of visits for responses 1–2/year, 3–5/year, 6–11/year, 1–3/month, 1–2/week, 3–5/week, almost every day, or never to be 1.5, 4, 8.5, 24, 78, 208, 365, and 0 visits, respectively. Figure 2 shows the distribution of number of visits by age and sex; in a multiple regression model, we found significantly (p<0.01) fewer visits to LPMs among younger adults but no significant differences by sex.

A total of 17.5% respondents reported that they had avoided visiting LPMs in the previous 7 days because of influenza A(H7N9), whereas 35.9% reported that they would support or strongly support permanent closure of LPMs. We used multivariable logistic regression to examine factors associated with avoiding LPMs or supporting closure of LPMs (Table 2) and found that more symptom-induced worry and higher perceived severity compared with seasonal influenza were associated with avoiding visiting LPMs. Younger age, lower educational attainment, and having visited LPMs >1 time in the preceding year were independently associated with a lower probability of support for permanent LPM closures.

**Table 2:** Factors affecting behavioral response to influenza A(H7N9) and support for permanent closure of LPMs in Hong Kong, 2013*

Characteristic	Odds ratio (95% CI)	
	Avoided visiting LPMs in previous 7 d because of influenza A(H7N9)	Would support permanent closure of LPMs
Sex		
M	Reference	Reference
F	1.07 (0.75–1.53)	1.22 (0.92–1.63)
Age, y		
18–34	Reference	Reference
35–54	0.89 (0.55–1.46)	2.28 (1.53–3.40)
≥55	0.81 (0.48–1.36)	2.87 (1.86–4.45)
Educational attainment		
Primary or below	Reference	Reference
Secondary	1.63 (0.92–2.89)	1.76 (1.17–2.65)
University or above	1.65 (0.88–3.09)	2.99 (1.88–4.76)
Visited LPM >1 time in previous year		
No	Reference	Reference
Yes	1.15 (0.78–1.68)	0.60 (0.44–0.83)
Median State Trait Anxiety score (*5*)		
<1.7	Reference	Reference
>1.7	1.03 (0.72–1.46)	0.95 (0.72–1.25)
Self-perceived risk for infection with influenza A(H7N9)†		
Low	0.68 (0.42–1.11)	0.95 (0.64–1.42)
Evens	Reference	Reference
High	0.95 (0.49–1.84)	1.51 (0.87–2.63)
Self-perceived risk for infection with influenza A(H7N9) compared with other persons‡		
Low	1.22 (0.74–1.99)	0.93 (0.62–1.38)
Evens	Reference	Reference
High	1.97 (0.51–7.61)	0.52 (0.13–1.98)
Symptom-induced worry§		
Less	1.01 (0.62–1.64)	0.87 (0.61–1.24)
As usual	Reference	Reference
More	2.00 (1.34–2.98)	1.08 (0.78–1.48)
Perceived severity compared with seasonal influenza¶		
Less	1.22 (0.53–2.79)	1.01 (0.59–1.73)
Same	Reference	Reference
More	2.31 (1.33–3.99)	1.28 (0.87–1.87)
Perceived severity compared with SARS¶		
Less	0.84 (0.52–1.36)	0.89 (0.61–1.29)
Same	Reference	Reference
More	0.91 (0.48–1.73)	0.88 (0.52–1.48)
Perceived severity compared with influenza A(H5N1)¶		
Less	1.29 (0.73–2.26)	0.76 (0.49–1.17)
Same	Reference	Reference
More	1.06 (0.70–1.60)	0.93 (0.66–1.31)

*Odds ratios adjusted for all variables shown. LPMs, live poultry markets; SARS, severe acute respiratory syndrome. †Respondents were asked, “How likely do you think it is that you will contract H7N9 avian flu over the next 1 month?” Low indicates the answers Never/Very unlikely/Unlikely; evens, the answer Same (50% probability); and high, the answers Likely/Very likely/Certain. ‡Respondents were asked, “What do you think are your chances of getting H7N9 avian flu over the next 1 month compared to other people outside your family of a similar age?” Low indicates the answers Not at all/Much less/Less; evens, the answer Same (50% probability); and high, the answers Likely/Very likely/Certain or More/Much more/Certain. §Respondents were asked, “If you were to develop flu-like symptoms tomorrow, would you be…” followed by several choices. Low indicates the answers Not worried at all/Much less worried than normal/Worried less than normal; as usual, the answer About same; and more, the answers Worried more than normal/Worried much more than normal/Extremely worried. ¶Respondents were asked, “How is the severity of infection with H7N9 avian influenza compared to seasonal influenza, SARS, or H5N1 avian influenza?” Lower indicates the answers A little lower/Much lower; same, the answer Same; and higher, the answers Much higher/A little higher.

## Conclusions

Results from previous studies and our surveys indicate that exposure to poultry measured by LPM visits among the Hong Kong population has declined since 2006 (*7*) and is lower than that for cities in southern China (*9*–*11*). Contact with live poultry or visiting LPMs was reported by most persons with confirmed influenza A(H7N9) in China (*2*). Four recently reported influenza A(H7N9) cases in Hong Kong were suspected to be imported; all 4 patients reported travel to Shenzhen, the city bordering Hong Kong, which reported 17 new cases during January 1–February 9, 2014. One of the patients in Hong Kong bought live poultry from an LPM in Shenzhen, where 2 LPMs subsequently yielded environmental specimens testing positive for influenza A(H7N9) virus (*12*).

We previously reported that LPM closure substantially reduced the risk for human infection with influenza A(H7N9) virus in mainland China (*3*). Control measures in LPMs in Hong Kong have become increasingly stringent during the past decade, and the current policy banning any overnight stay of live poultry in LPMs has substantially decreased avian influenza virus prevalence among poultry (*13*). This policy might also contribute to the low perceived risk for infection and low levels of symptom-induced worry observed in this study (Table 1).

Our study has limitations. Because participants were recruited on the basis of randomly selected telephone numbers, respondents might not represent the general population in Hong Kong, despite weighting of the sample by age and sex. Responses in the survey were self-reported and might be subject to response biases, including social desirability bias. We also used contact history in the previous year to measure respondents’ live poultry exposure, which could be subject to recall bias.

In conclusion, our survey found generally low anxiety levels among the population in Hong Kong related to the threat of influenza A(H7N9). A higher level of symptom-induced worry and higher perceived severity of influenza A(H7N9) compared with seasonal influenza were associated with avoidance of LPMs. Permanent closure of LPMs is being considered in Hong Kong, but our results suggest that obtaining support from the public might be difficult, particularly among younger adults and adults with lower educational attainment.

Technical Appendix Characteristics of persons surveyed during influenza A(H7N9) epidemic, Hong Kong, April and December 2013
