# Supplementary material for: Responses to Threat of Influenza A(H7N9) and Support for Live Poultry Markets, Hong Kong, 2013
Source: Emerg Infect Dis. 2014 May;20(5):882–6. doi: 10.3201/eid2005.131859 (PMC4012820; doi:10.3201/eid2005.131859)
Supplement: Technical Appendix — Characteristics of persons surveyed during influenza A(H7N9) epidemic, Hong Kong, April and December 2013 [file 13-1859-Techapp-s1.pdf]

Article DOI: http://dx.doi.org/10.3201/eid2005.131859

Responses to Threat of Influenza A(H7N9) and Support for Live Poultry Markets, Hong Kong, 2013

Technical Appendix

Technical Appendix Table. Characteristics of persons surveyed during influenza A(H7N9) epidemic, Hong Kong, April and December 2013

Characteristic	First survey, April 10–13 and 25–27, 2013 (n = 1,556)	Second survey, December 4–8, 2013 (n = 1,000)
Sex		
F	62.8%	62.6%
M	37.2%	37.4%
Age group, y		
18–34	25.4%	24.2%
35–54	36.5%	33.1%
>65	38.1%	42.7%
Education*		
Primary or below	13.9%	15.1%
Secondary	48.5%	44.8%
Tertiary or above	37.5%	40.1%
Place of birth*		
Hong Kong	69.0%	67.6%
Other places	31.0%	32.4%
Marital status*		
Single	28.9%	27.6%
Married/formerly married	71.1%	72.4%
Monthly household income, HK$*†		
<$10,000	11.7%	12.5%
$10,000–$20,000	23.4%	22.0%
$20,000–$40,000	38.1%	35.0%
≥$40,000	26.8%	30.6%

*Proportions for these demographic variables were weighted by age and sex.

†US$1 = HK$7.8
